# Effect of Methods of Biosilicate Microparticle Application on Dentin Adhesion

**DOI:** 10.3390/dj7020035

**Published:** 2019-04-01

**Authors:** Michelle Alexandra Chinelatti, Egle Leitão Santos, Camila Tirapelli, Fernanda Carvalho Panzeri Pires-de-Souza

**Affiliations:** Department of Dental Materials and Prosthodontics, Ribeirão Preto School of Dentistry, University of São Paulo, Av. do Café s/n, Monte Alegre, Ribeirão Preto, São Paulo 14040-904, Brazil; egle.santos@usp.br (E.L.S.); catirapelli@forp.usp.br (C.T.); ferpanzeri@usp.br (F.C.P.P.-d.-S.)

**Keywords:** dentin, surface treatment, micro-tensile, hybrid layer, bioglass

## Abstract

Restorative procedures associated with bioglasses have shown to be a strategy to satisfy the contemporary concept of minimally invasive dentistry. Thus, the aim of this study was to evaluate bond strength to dentin treated by two different methods of biosilicate microparticle application. Dentin surfaces from 30 sound human molars were exposed and randomly assigned into three groups (*n* = 10) according to the surface treatment: (1) blasting with biosilicate microparticles (distance = 1 cm/pressure = 5 bar/time = 1 min); (2) 10% biosilicate microparticles paste; and (3) control (no treatment). After, dentin surfaces were restored with self-etch adhesive (Adper Easy Bond) and nanofilled composite (Filtek Z350). Specimens were sectioned perpendicularly to the adhesive interface to obtain sticks (cross-section area = 1 mm^2^), which were submitted to microtensile test (0.5 mm/min; 50 kgf). Data were analyzed by ANOVA and Tukey’s test (α = 5%). Dentin/adhesive interfaces were morphologically analyzed by scanning electron microscopy (SEM). Data analysis showed that biosilicate-treated groups reached similar results (*p* > 0.05) and both of them demonstrated higher values (*p* < 0.05) than control group. SEM micrographs revealed hybridization with clear resin tags and no separation between resin-dentin adhesive interfaces. Within the limitations of this study, surface treatment with biosilicate positively influenced the adhesion to dentin and does not alter the morphology of the adhesive interface.

## 1. Introduction

Modern operative dentistry is moving to a minimally invasive approach based on a philosophy that integrates prevention, remineralization, and minimal intervention for the placement and replacement of restorations preferably performed with adhesive restorative materials [1,2]. When a caries lesion has advanced to surface cavitation and the dentin is involved, its affected layer can be remineralized [3]. For this purpose, bioactive materials, such as bioglasses, can be applied on dental substrate to stimulate its remineralization [4,5,6]. In general, bioglasses react with hard tissues, favoring hydroxyapatite formation and tissue remineralization [7]. The combination of procedures performed with bioglasses has proved to be an operative strategy to satisfy the contemporary concept of minimally invasive dentistry [4,8,9]. Research on bioglasses led to the development of a new fully crystalline glass ceramic, named Biosilicate [10,11]. Biosilicate is able to bind chemically to bone and dental tissues through a carbonated hydroxyapatite layer formation whose chemical composition and structure are similar to the mineral phase of biological hard tissues [11]. When in contact with dentin, biosilicate particles react rapidly with peritubular and intratubular dentin and induce hydroxyl carbonate apatite deposition in open dentinal tubules [12,13]. Moreover, particles adhered to tissues constantly release calcium and phosphate ions, increasing the local pH and favoring the process of dental remineralization [12,13,14]. In order to reach the treatment objective of minimally invasive dentistry, together with a remineralizing approach, it is necessary to maximize the integrity of the dentin bonding interface by use of adhesive restorative materials [1,2]. In this way, applying bioglasses as remineralizing agents on dentin could promote alterations in its structure and influence the adhesion process. Nevertheless, previous research [8,9,15,16] on dentin adhesion showed that surface treatment with bioactive glasses, including biosilicate, did not diminish the adhesion of tested restorative materials. Moreover, it has also been demonstrated the advantages of the clinical use of blasting systems with bioactive glasses for dentin treatment prior restorative procedures with adhesive materials [17,18,19]. However, there are no reported studies using blasting system as a method of biosilicate microparticle application for dentin treatment previously to an adhesive restorative procedure.

Thus, the purpose of this study was to evaluate the effect of two different methods of biosilicate microparticle application on adhesion to dentin and to analyze the micromorphological aspects of the resultant adhesive interfaces. The null hypothesis was that the tested methods of microparticle application would not influence adhesion to dentin and the micromorphology of the adhesive interfaces. 

## 2. Materials and Methods

### 2.1. Experimental Outline

The study factor was *dentin surface treatment* at three levels: (1) Blasting with biosilicate microparticles; (2) biosilicate microparticle paste; and (3) no treatment (control). The study sample was composed of 30 dentin surfaces randomly assigned into three groups (*n* = 10). The quantitative response variable was bond strength in MPa. The morphology of the dentin/adhesive interfaces were observed by scanning electron microscope (SEM). Table 1 describes the characteristics of the materials used in this study.

#### Ethical Aspects

After approval by the Research Ethics Committee (CAAE 08122612.9.0000.5419; 19 June 2014) of the Ribeirão Preto School of Dentistry (FORP) of the University of São Paulo (USP), 39 third sound human molars were selected from the Human Teeth Bank of FORP.

### 2.2. Selection and Preparation of Teeth

The teeth were cleaned using periodontal curettes and pumice slurry in a slowly rotating rubber cup. Then, they were observed by a stereoscopic loupe, and those presenting cracks or structural anomalies were discarded. The radicular portion of the teeth was embedded in polyester resin through a PVC ring to facilitate the removal of the occlusal surface using a diamond disc set in a metallographic cutter, thereby exposing the surface of coronal dentin. Each dentin surface was then ground using a series of silicon carbide discs (#320, 600, 1000, and 1200) under water cooling. The exposed dentin surfaces were examined in a stereoscopic microscope (EK3ST, Eikonal do Brasil, São Paulo, SP, Brazil), at 30× magnification, to make sure no enamel was left. The teeth were stored in distilled water for 24 h at 4 °C, after which they were randomly assigned into three groups (n = 10), according to their respective surface treatment.

### 2.3. Dentin Surface Treatments

(1) Biosilicate microparticle blasting consisted of blasting the dentin surface with biosilicate powder using a hand piece (MicroJet, Bio-Art, São Carlos, SP, Brazil), under 5 bar (500 MPa) for 1 min, at a distance of 1 cm from the dentin surface [18]. 

(2) Biosilicate paste: a 10% biosilicate paste was prepared immediately before application by adding 1 mL of distilled and deionized water in microtubes containing 0.1 g of biosilicate powder. The paste was first scrubbed onto the dentin surface for 30 seconds using a microbrush and, then, it remained still for five minutes [14]. Afterwards, the material was carefully removed with absorbent paper.

(3) Control: No surface treatment.

### 2.4. Restorative Procedure

After each treatment, dentin surfaces were restored. The bonding agent (Adper Easy Bond Self-etch Adhesive—3M Dental Products, St. Paul, MN 55144-1000, USA) was applied following the manufacturer’s instruction and light-cured for 20 seconds using an LED curing unit (Flashlite 1401 LED Discus Dental, Inc., Culver City, CA, USA). The delimited dentin surfaces (5 × 5 mm) were restored with a nanofilled composite resin (Filtek Z-350—3M Dental Products, St. Paul, MN 55144-1000, USA) according to the incremental insertion technique (2 mm), resulting in a final 4-mm-thick layer. Each layer (2 mm) was light-cured for 40 seconds. Finally, every side of the restoration was additionally light-cured for 20 seconds. The intensity of the light curing unit was measured periodically, using a radiometer (RD-7, Ecel Ind. e Com. Ltda, Ribeirão Preto, SP, Brazil) at an average interval of 1100 mW/cm². The specimens were then stored in 37 °C distilled water for 24 h.

### 2.5. Microtensile Bond Strength Test

Each specimen was sectioned perpendicularly to its bonding interface in mesial-distal and buccal-lingual directions using a diamond disc coupled to a low-speed cutting machine under water cooling. Stick-shaped specimens, with a cross-section area of approximately 1.0 mm², were obtained and measured with a digital caliper to determine their individual sectioned areas, since the microtensile bond strength values were calculated by dividing the load at failure by the cross-sectional bonded area. After 24 h storage in distilled water at 37 °C, each stick-shaped specimen was positioned and fixed with cyanoacrylate adhesive (Super Bonder Gel, Henkel Ltda., São Paulo, SP, Brazil) into a specific metallic device for microtensile bond strength test in a universal testing machine (EMIC Equipamentos e Sistemas de Ensaio Ltda., São José dos Pinhais, PR, Brazil) at 0.5 mm/min speed and 50 kgf load. For comparison, the average of the tested sticks (around five sticks) was used as the result of each specimen. 

### 2.6. Statistical Analysis

The bond strength values were analyzed using ANOVA and Tukey post-hoc by statistical software (GraphPad Prism 4.0^®^—GraphPad Software, Inc., La Jolla, CA, USA) at a 5% level of significance.

### 2.7. Failure Mode Analysis

Debonded dentin surfaces were analyzed under a stereomicroscope at 100× magnification (Olympus SZ40, Tokyo, Japan) to observe the failure modes. The failure modes were classified as adhesive (A: failure at the resin-dentin interface), cohesive (C: failure exclusively within dentin or composite resin), or mixed (M: presence of both adhesive and cohesive failures). 

### 2.8. SEM Analysis of the Adhesive/Dentine Interfaces

To analyze the morphology of the adhesive/dentine interfaces, nine additional dentin surfaces (n = 3) were prepared and restored as previously described. The specimens were sectioned perpendicularly to the bonding interface, yielding four 1-mm-thick sections. These sections were then polished manually using a series of silicon carbide discs (#600, 800, 1000, 1200, and 2000) under water cooling, and then with aluminum paste (Struers ApS, Ballerup, Denmark) on a silk cloth. The polished sections were prepared for SEM analysis, as follows: 12 h of immersion in glutaraldehyde solution (2.5%) in 0.1 M sodium cacodylate buffer, 7.4 pH (Merck KGaA, Darmstadt, Germany) at 4 °C. After immersion period, the specimens were rinsed in distilled water for 3 min, and, then, immersed in distilled water for one hour, changing the water every 20 min. Afterwards, the surfaces to be analyzed were treated with 37% phosphoric acid for 10 s, washed, immersed in distilled water, and placed in an ultrasound machine (T-1449-D, Odontobrás, Ribeirão Preto, SP, Brazil) for 10 min to eliminate any possible remaining residues. This was followed by dehydration through immersion in a series of ethanol concentrations (Labsynth Ltda., Diadema, SP, Brazil): 25% (20 min), 50% (20 min), 75% (20 min), 95% (30 min), and 100% (60 min). Finally, the sections were placed in a HMDS solution for another 10 min (Merck KGaA, Darmstadt, Germany), dried with absorbent paper towels, glued onto stubs, and gold coated. During the SEM (Philips XL-30 FEG. FEI Company, Eindhoven, Netherlands) analysis, scanning was performed in a systematic pattern over the entire adhesive/dentine interfaces to record and transmit images at different magnifications (1000–4000×). The morphology of the adhesive/dentine interface was observed to identify the formation of hybrid layer, based on an analysis of its integrity, homogeneity and thickness; uniformity of size and arrangement of tags; as well as the presence of bioglass microparticles inside dentinal tubules. 

## 3. Results

### 3.1. Microtensile Bond Strength Test

Table 2 lists the mean values of bond strength to dentin as a function of surface treatments. An analysis of the results indicated that the groups that underwent biosilicate surface treatment showed higher adhesive strength than the control group (*p* < 0.05). However, the surface treatments by biosilicate blasting and biosilicate paste showed no statistically significant difference (*p* > 0.05).

### 3.2. Failure Mode Analysis

Table 3 describes the distribution, in percentage, of failure modes found in the specimens of all groups. The analysis revealed that the majority of failure mode exhibited in all groups was classified as mixed, followed by adhesive and cohesive, sequentially.

### 3.3. SEM Analysis of Adhesive Interfaces

**Figure 1:**
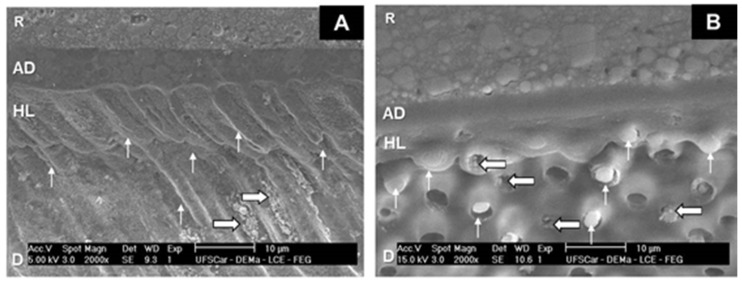
SEM micrographs of Group 1. (**A**) 2000×. Note the hybrid layer (HL) at the dentin (D)/adhesive (AD)/composite resin (R) interface, with resinous tags (thin arrows). Thick arrows indicate the deposition of biosilicate microparticles in dentin tubules. (**B**) 2000×. Hybrid layer (HL) at the dentin (D)/adhesive (AD)/composite resin (R) interface; thin arrows: resinous tags; thick arrows: biosilicate microparticles in dentin tubules.

**Figure 2:**
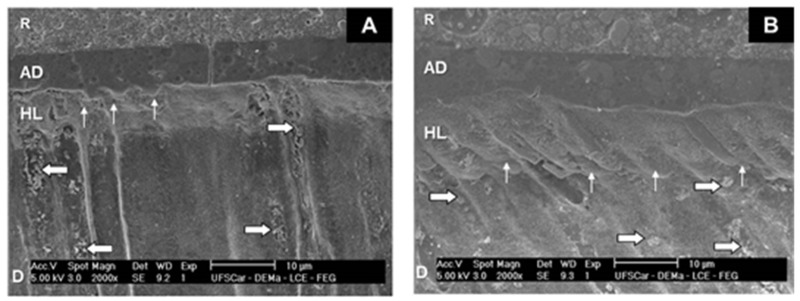
SEM micrographs of Group 2. (**A**) 2000×. Note the hybrid layer (HL) at the dentin (D)/adhesive (AD)/composite resin (R) interface, with resinous tags (thin arrows), and biosilicate microparticles (thick arrows). (**B**) 2000×. Hybrid layer (HL) at the dentin (D)/adhesive (AD)/composite resin (R) interface; thin arrows: resinous tags; thick arrows: biosilicate microparticles.

**Figure 3:**
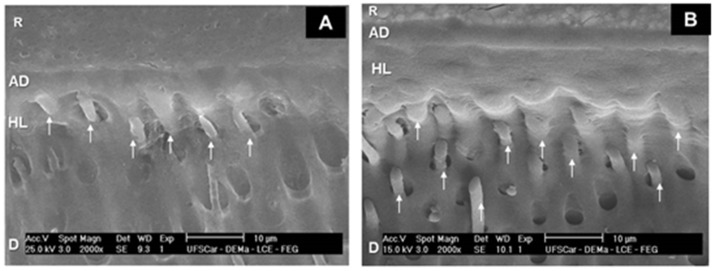
SEM micrographs of Group 3. (**A**) 2000×. Note the uniformity of hybrid layer (HL) at the dentin (D)/adhesive (AD)/composite resin (R) interface, with resinous tags (thin arrows). (**B**) 2000×. Hybrid layer (HL) at the dentin (D)/adhesive (AD)/composite resin (R) interface, and resinous tags (thin arrows).

The micrographs of Group 1 (Figure 1) revealed the deposition of microparticles in dentinal tubules underneath the homogeneous hybrid layer, as well as the presence of resinous tags arranged at the adhesive/dentine interface. The adhesive/dentine interfaces of Group 2 (Figure 2) showed the same hybrid layer pattern, albeit with a smaller amount of microparticles. All adhesive interfaces of control group also showed a uniform hybrid layer (Figure 3).

## 4. Discussion

Based on the results of this study, the null hypothesis was rejected. Either methods of application of biosilicate microparticles had a positive influence on the bond strength values to dentin without altering the adhesive interface morphology. In this study, both the biosilicate groups showed higher values of microtensile bond strength than the control group, corroborating with previous studies [6,7,8,9,10] on dentin surface treatment with biosilicate microparticles. The application of biosilicate microparticles on dentin induces the deposition of carbonated hydroxyapatite, which leads to the formation of a mineralized surface layer [12,13]. This layer adhering to dentin constantly releases calcium and phosphate ions that are present in the composition of microparticles, becoming possible adjuvant factors for tooth remineralization [14,15,16]. Moreover, a previous study [18] suggested that blasting with bioactive glasses could increase the durability of dentin bonds, could induce dentin remineralization, and could enhance its repair capacity. This finding is in line with the current concept of minimally invasive operative treatments [1,2]. Results from previous studies [16,17] employing biosilicate microparticle suspension as a dentin pre-treatment did not show a negative influence on the bond strength of total-etch and self-etch adhesive systems. Despite this fact, the self-etching strategy was adopted in this study because their use on dentinal tissue is currently preferred [20,21]. The main disadvantage of total-etch adhesives is their susceptibility to the complete filling of the interfibrillar spaces by resin monomers thus resulting in areas of exposed demineralized dentin within the bonded interface. This incomplete infiltration of resin monomers network may lead to interfacial hydrolysis and postoperative sensitivity [20,21]. Moreover, the bonding effectiveness of self-etch adhesives has been widely reported [20,21] and it has been attributed to their ability to demineralize and infiltrate the dentin surface simultaneously to the same depth, preventing incomplete penetration of the adhesive into the exposed collagen network. The presence of smear-layer and smear-plugs during the bonding procedure prevents fluid movement through dentin, acting as an important barrier to dentin permeability [20,21]. The formulation of the self-etch adhesive system used includes a carefully balanced combination of phosphoric acid esters, water and methacrylates in order to optimize stability. In addition, bonded nanosilica fillers give enhanced bond strength [22]. As biosilicate was applied before adhesive application, it may have contributed to the deposition of mineral microparticles on dentin surface [12,13]. This augmented mineral content would act as a receiver for additional chemical bonding with a functional monomer (functional methacrylate copolymer of polyacrylic and polylactic acids) contained in the adhesive composition [22]. A hydroxyapatite chemical bond may have been formed by phosphoric acid esters and functional adhesive copolymer, creating a complex with calcium ions similar to those created with glass ionomer cements on dentin conditioned with polyacrylic acid [22]. In addition, it has been suggested that the presence of filler particles favors the development of a uniform adhesive layer and stabilizes the hybrid layer [22]. A possible explanation for this more stable adhesive layer is that it may be due to the chemical bond created by the supposed mineral precipitation on the resin-dentin interface when bioglasses are used [23]. In the SEM micrographs of biosilicate groups, it is possible to observe the presence of microparticles, mainly in the blasting group, suggesting that biosilicate microparticles were incorporated in dentinal tubules and on the dentin surface. This statement is confirmed by the results of previous studies [15,16] which verified the presence of biosilicate microparticles by backscatter method. Even so, deposition of biosilicate microparticles did not interfere with the morphological aspects of the resultant adhesive/dentin interface, taking into account occurrence of hybridization and uniform tags for all analyzed groups.

Within the limitations of the present study, the use of biosilicate microparticles as a dentin treatment positively influenced the adhesive bond strength. Nonetheless, future in vivo and clinical studies still must be conducted in order to reach a biosilicate effect on the longevity of the adhesive interface, as well as its scientific relevance and wide range of applications as a therapeutic option in different dentin conditions, such as pathological or sclerotic dentin.

## Figures and Tables

**Table 1:** Materials used in this study.

Material	Composition	Manufacturer
Biosilicate microparticles	Fully crystallized glass-ceramic of the Na_2_O-CaO-SiO_2_-P_2_O_5_ system, with additions of Li_2_O and K_2_O; 1–10 µm.	Vitrovita, São Carlos, SP, Brazil
Filtek™ Z350—composite resin	Inorganic Fillers: 78.5% by weight. Non-agglomerated/non-aggregated 20 nm silica filler, non-agglomerated/non-aggregated 4–11 nm zirconia filler, and aggregated zirconia/silica, cluster filler (20 nm silica and 4–11 nm zirconia). Resins: Bis-GMA *, UDMA **, Bis-EMA ***, TEGDMA ^##^.	3M ESPE Dental Products, St. Paul, CA, USA
Adper™ Easy Bond Self-Etch Adhesive	2-Hydroxyethyl methacryate (HEMA), Bis-GMA, methacrylated phosphoric esters, 1,6 hexanediol dimethacrylate, methacrylate functionalized, polyalkenoic acid (Vitrebond™ Copolymer), 7nm silica filler, ethanol, water, camphorquinone, stabilizers.	3M ESPE Dental Products, St. Paul, CA, USA

* Bis-GMA: bisphenol A diglycidyl ether dimethacrylate; ** UDMA: urethane dimethacrylate; *** Bis-EMA: ethoxylated bisphenol-A dimethacrylate; ^##^ TEGDMA: triethylene glycol dimethacrylate.

**Table 2:** Mean values (MPa) and standard deviations (±SD) of microtensile bond strength to dentin as a function of surface treatments.

Surface Treatment (Group)	MPa (±SD)
Biosilicate microparticles blasting (Group 1)	28.4 (±2.3) ^a^
Biosilicate microparticles paste (Group 2)	27.7 (±2.1) ^a^
Control—No treatment (Group 3)	22.3 (±1.4) ^b^

Different superscript letters (a and b) indicate statistically significant difference (*p* < 0.05).

**Table 3:** Distribution (%) of failure modes of each group.

Group	Adhesive	Cohesive	Mixed
**1**	21	8	71
**2**	20	11	69
**3**	18	17	65
